# An unforeseen polymorph of coronene by the application of magnetic fields during crystal growth

**DOI:** 10.1038/ncomms11555

**Published:** 2016-05-10

**Authors:** Jason Potticary, Lui R. Terry, Christopher Bell, Alexandros N. Papanikolopoulos, Peter C. M. Christianen, Hans Engelkamp, Andrew M. Collins, Claudio Fontanesi, Gabriele Kociok-Köhn, Simon Crampin, Enrico Da Como, Simon R. Hall

**Affiliations:** 1 Complex Functional Materials Group, School of Chemistry, University of Bristol, Bristol BS8 1TS, UK; 2 School of Physics, HH Wills Physics Laboratory, Tyndall Avenue, Bristol BS8 1TL, UK; 3 High Field Magnet Laboratory (HFML-EMFL), Radboud University, Toernooiveld 7, 6525 ED Nijmegen, The Netherlands; 4 Bristol Centre for Functional Nanomaterials, HH Wills Physics Laboratory, Tyndall Avenue, Bristol BS8 1TL, UK; 5 Department of Physics, University of Bath, Claverton Down, Bath BA2 7AY, UK; 6 Dipartimento di Ingegneria Enzo Ferrari, Universita' di Modena e Reggio Emilia, Via Vivarelli 10, 41125 Modena, Italy; 7 Department of Chemistry, University of Bath, Claverton Down, Bath BA2 7AY, UK

## Abstract

The continued development of novel drugs, proteins, and advanced materials strongly rely on our ability to self-assemble molecules in solids with the most suitable structure (polymorph) in order to exhibit desired functionalities. The search for new polymorphs remains a scientific challenge, that is at the core of crystal engineering and there has been a lack of effective solutions to this problem. Here we show that by crystallizing the polyaromatic hydrocarbon coronene in the presence of a magnetic field, a polymorph is formed in a β-herringbone structure instead of the ubiquitous γ-herringbone structure, with a decrease of 35° in the herringbone nearest neighbour angle. The β-herringbone polymorph is stable, preserves its structure under ambient conditions and as a result of the altered molecular packing of the crystals, exhibits significant changes to the optical and mechanical properties of the crystal.

## Abstract (web summary)

Polymorphism, the presence of different crystal structures of the same molecular system, provides an opportunity to discover new phenomena and properties. Here, the authors crystallize coronene in the presence of a magnetic field, forming a different polymorph, which remains stable under ambient conditions.

## Graphical abstract

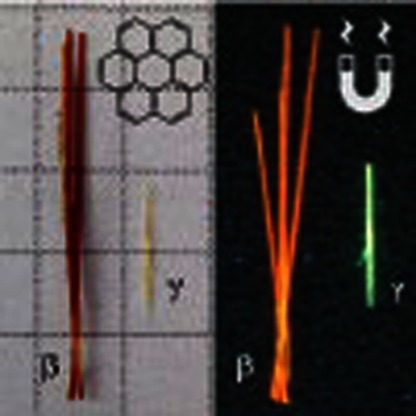

The ability to discover new phenomena and properties in materials depends on our ability to synthesize new structures. The discovery of new structures, however, should rely on variables that transcend the typical thermodynamic parameters of temperature and pressure. In the field of functional molecular materials, polymorphism, the presence of different crystal structures of the same molecular system, can be an opportunity to discover novel phenomena^1^ and tune properties^2^. For example, charge carrier mobility in organic semiconductors can be increased by crystallizing molecules under pressure, resulting in shorter intermolecular distances favourable for transport^3^. One successful tool at the scientist's disposal to control crystal structure has been to perform crystallization in the presence of electric fields. This is a well-known process and is used extensively to prepare nonlinear optical materials through electric poling. Crystal growth in the presence of an external magnetic field is much less explored, although it has been proven to be efficacious in the melt texturing of alloys^4^ or in cases where controlled convection of the crystallizing solution is required, for example, in the growth of high-quality protein crystals^5^, chiral aggregates^6^ or in the alignment of liquid crystal and block copolymer arrays^7^,^8^. A magnetic field has even been demonstrated to be able to separate polymorphs of crystals post-synthetically^9^ and in one case to preferentially nucleate a monoclinic form of terpyridine^10^. Although it is known that magnetic forces can have an effect on solidification and subsequent physical behaviour in crystals, their use to create previously unknown polymorphs in single crystals has never been reported.

Polyaromatic hydrocarbons (PAHs) are commonly researched molecules due to their rigid planar structure, high stabilities and characteristic optical and electronic behaviour^10^,^11^. As molecular solids, PAHs crystallize in four basic structure types according to well-defined geometric and energetic considerations^12^. These are the herringbone structure, the gamma-herringbone (γ-) structure, the sandwich-herringbone (SHB) structure and the beta-herringbone (β-) structure (Supplementary Fig. 1). Comprehensive studies of PAHs have shown that the adoption of one of the four structure types depends ultimately on the relative strength of nonbonded C⋯C and C⋯H interactions^12^,^13^,^14^. Polymorphism in PAHs has been demonstrated previously by growing thin films on substrates, where variations in processing conditions direct the formation of different polymorphs^15^,^16^, although in single crystals at ambient pressure, polymorphism in PAHs is rare with perylene and pyrene being two notable cases exhibiting both herringbone and SHB polymorphs^17^. In terms of applications, single crystals of PAHs are to be desired, however, as they have distinct physical advantages over thin-film versions of the same material. Most importantly, for conductive applications such as transistors, or optical applications in solar cells, single crystals typically have higher carrier mobilities than their thin-film analogues^18^,^19^, which have to be defect free to achieve mobilities of the same order of magnitude.

Here we show that by crystallizing the PAH coronene in the presence of a magnetic field, it can be made to form as a new β-herringbone polymorph instead of the ubiquitous γ-herringbone form, with a change as large as 35° to the herringbone nearest neighbour angle. The β polymorph is stable and can preserve its structure in ambient conditions and zero magnetic field. Dispersion corrected density functional theory (DFT-D) calculations indicate that the new form is energetically favoured at low temperature. Furthermore, we demonstrate how the new supramolecular structure generates remarkable changes of the electronic, optical and mechanical properties in the crystal.

## Results

### Crystal growth of a new polymorph

**Figure 1:**
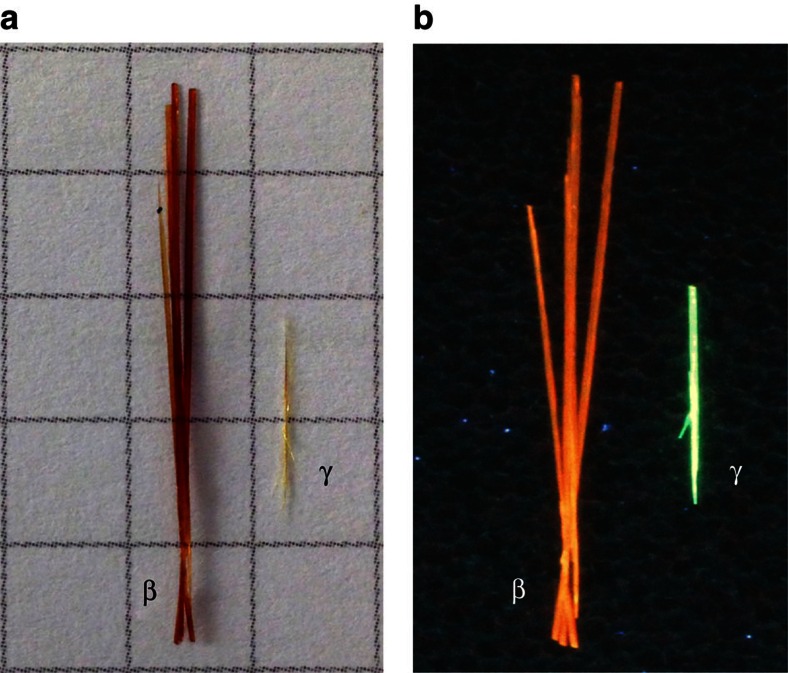
Optical images of the β- and γ- polymorphs of coronene. (**a**) in daylight and (**b**) under ultraviolet (*λ*=365 nm) illumination to show fluorescence. The squares on the grid in **a** are 0.5 × 0.5 cm^2^.

**Figure 2:**
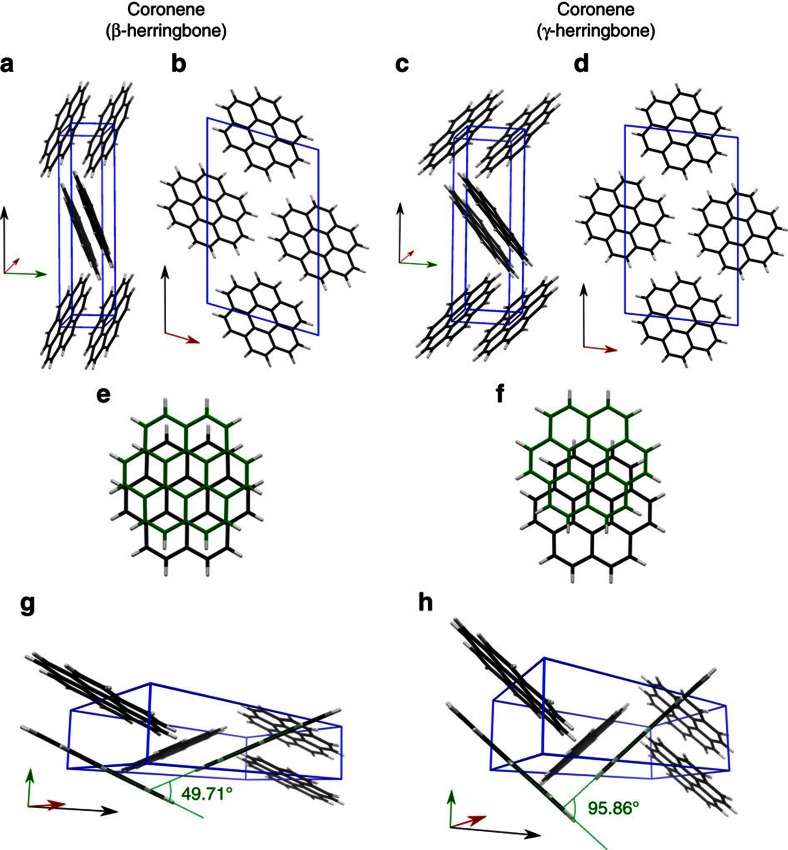
Representation of the β- and γ- polymorphs of coronene. Differing perspectives of both unit cells (blue boxes) viewed slightly offset from along the *a*-axis (**a** and **c**) and along the *b*-axis (**b** and **d**). The relative shift of the molecules along the stacks are shown for β- (**e**) and γ- (**f**). (**g**) and (**h**) show an orientation of the unit cell clearly demonstrating the difference in nearest neighbour angle between the two polymorphs. Red green and black arrows indicate the direction of the *a*-, *b*- and *c*-axis respectively.

Coronene is a PAH composed of six aromatic rings arranged in a planar discoidal geometry (Supplementary Fig. 2). The high molecular symmetry (D_6h_) and 24 electron π-system has made coronene an ideal model system for the study of graphene, due to it being large enough to display exotic electronic behaviour, but not so large that contortion becomes a complicating factor^20^. Centimetre-long crystals of coronene (typically ∼0.75 cm) were grown from a supersaturated solution of the molecules in toluene cooled slowly (0.04 K min^−1^) from 328 K to 298 K over a period of 12 h (Fig. 1a, yellow crystal labelled ‘γ'). The very slow cooling rate was chosen to eliminate changes in solution shearing which have been shown to influence nucleation and growth with concomitant changes to the resultant crystal structure^21^. Single crystal X-ray diffraction (XRD) of these crystals indicated that the structure is the conventional γ-polymorph; *a*=10.02 Å, *b*=4.67 Å, *c*=15.60 Å, *β*=106.7°, *Z*=2, space group P2_1_/n, with an inter-planar distance (*d*_*π*_) of 3.43 Å, which is consistent with parallel π-stacking^12^ (Fig. 2a,b,e,g).

In the presence of an external magnetic field of 1 Tesla, however, significantly longer (typically ∼2.5 cm) coronene crystals were grown from the same supersaturated solution and exhibit a different colour to normal γ-coronene crystals (Fig. 1a orange crystal labelled ‘β'). Single crystal XRD indicates that the unit cell of these crystals are consistent with a new β-coronene structure; *a*=10.39 Å, *b*=3.84 Å, *c*=17.23 Å, *β*=96.24°, *Z*=2, space group P2_1_/n and an inter-planar distance (*d*_*π*_) of 3.48 Å (Fig. 2c,d,f,h). As a previously unreported polymorph of coronene, the crystallographic data has been lodged with the Cambridge Crystallographic Data Centre (deposition number CCDC 1409823).

**Figure 3:**
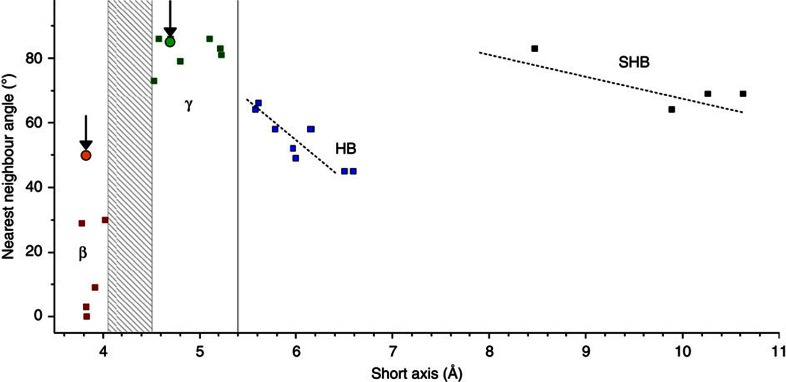
Grouping of PAHs into structure types. Plotted on the graph are PAHs that adopt the herringbone structure (blue squares), the gamma-herringbone (γ-) structure (green squares), the SHB structure (black squares) and the beta-herringbone (β-) structure (red squares), according to the crystallographic short axis and nearest neighbour herringbone angle. The positions of both γ- and β-coronene polymorphs are indicated by circles and marked with arrows. Adapted from Desiraju and Gavezzotti^12^, in which the names of the crystals corresponding to all of the marked squares can be found. Reproduced with permission of the International Union of Crystallography, http://journals.iucr.org/.

By comparing the two crystal forms in Fig. 2c, it can be seen that the short axis, *b*, is substantially decreased in length for the β-coronene crystallized in the magnetic field when compared with the γ-coronene. This new polymorph also has a significantly smaller nearest neighbour herringbone angle of 49.71° compared with 95.86° in γ-coronene. The identification of the new crystal polymorph as a β-structure can be made by reference to a plot of the inter-planar angle of nearest neighbour molecules versus the unit cell short axis^12^ (Fig. 3). From this, it can be seen that the new polymorph sits squarely with other PAHs identified as having the β-herringbone structure. Under an applied magnetic field of 1 T, the new β-coronene polymorph grows exclusively, is reproducible (>10 times to-date), stable under ambient conditions and also forms in the non-aromatic solvent hexane.

To determine the mechanism of crystal growth, we followed the inception of crystallization of coronene at 1 T and 0 T via *in situ* UV–vis spectrometry. At 358 nm, there are no peaks due to molecules of coronene in solution, nor in the solid state (as crystals). However, a peak will be observed due to the formation of nucleation clusters, which we determined via a single-point energy calculation using time-dependent self-consistent field density functional theory, using basis set 6-31G(D). Supplementary Figure 3 shows that there is a stark difference between the growth of coronene crystals under 1 T of applied field and at 0 T. At 1 T, there is suppression of nucleation which results in the crystallization of the least stable polymorph, that is, beta-coronene. This is entirely consistent with previously published work on the formation of meta-stable polymorphs and is epitomized in Ostwald's rules of stages^22^. Our experimental data therefore demonstrates that the magnetic field is achieving the effect of polymorphic control through suppression of nucleation.

As to the question of why an applied magnetic field of 1 T is having this effect in coronene and not in other molecules at this field strength, it is likely to be intimately linked to the magnetic susceptibility of the molecule. Based on inductively coupled plasma atomic absorption spectroscopy, we note that the level of magnetically active impurities such as cobalt, iron and nickel are at the parts per billion level (Co=0.138±0.98 ppb; Fe=33.81±0.56 ppb; and Ni=13.46±0.65 ppb), and so are unlikely to play any role in polymorph selection. Instead we note that coronene has a very high diamagnetic susceptibility (χ) of −243 × 10^−6^ emu mol^−1^ (refs 23, 24). Crystal growth performed at weaker field strengths of 0.2, 0.5 and 0.8 T resulted in crystals of the γ−polymorph (Supplementary Fig. 4), suggesting that 1 T is close to a threshold for energetic selection between the two forms. In the case of other PAHs such as pyrene, which do not have such a strong diamagnetic susceptibility, our attempts to control polymorphism at 1 T were unsuccessful. It would appear that for molecules with lower diamagnetic susceptibilities, higher fields would be required. Indeed, work reported in a thesis in 2013 has shown that a simple organic dye molecule with a lower diamagnetic susceptibility than coronene, namely an isoxazolone derivative, can be crystallized in a different known polymorph at fields >2.5 T (ref. 25).

### Optical behaviour

**Figure 4:**
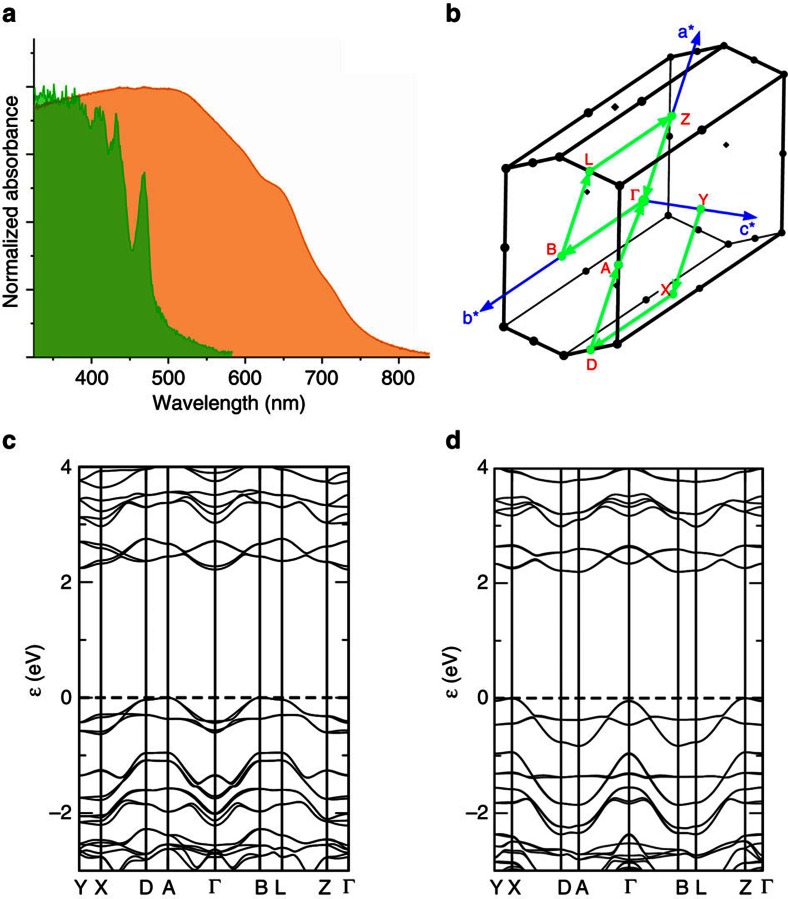
Electronic structure of γ- and β-coronene. (**a**) Absorption spectra of γ-coronene (green) and β-coronene (orange) single crystals. Unpolarized light was irradiated perpendicular to the *a–b* plane at room temperature; (**b**) Brillouin zone with reciprocal lattice vectors and high symmetry points; (**c**) and (**d**) band dispersion along high symmetry points in γ- and β-coronene, respectively.

Figure 1b shows optical images of the two different polymorphs taken under UV irradiation (λ=365 nm). The remarkably different colours of the fluorescence of γ-coronene and β-coronene crystals is further confirmation that the molecular packing in the crystal has been transformed, leading to altered electronic behaviour. To quantify this, we measured the absorption spectrum of two single crystals of the two polymorphs by shining unpolarized light perpendicular to the *a–b* plane of the crystals, that is, light propagation parallel to [001]. As shown in Fig. 4a (green plot), the γ-coronene single crystal is characterized by a first absorption resonance at 468 nm assigned to the free exciton in coronene, in agreement with previous studies^26^. The β-coronene spectrum is by stark contrast almost featureless, with an absorption onset at 780 nm and a maximum at ∼500 nm (Fig. 4a, orange plot). This is a remarkable change in the optical properties of this material. These data can be plotted as the extinction molar coefficient versus wavelength, to decouple any effects due to differences in the thickness of the crystals of the two polymorphs (50 microns and 76 microns for γ-coronene and β-coronene, respectively; Supplementary Fig. 5). From these spectra, it can be seen clearly that β-coronene is a more strongly absorbing polymorph than γ-coronene. Organic materials tend to be sensitive to a particular range of wavelengths, which can be seen in the features of the γ-coronene absorption spectrum and the lack of absorption of wavelengths >583 nm. In β-coronene, however, the crystal strongly absorbs over a wide band of radiation, from the UV into the near-IR (320–847 nm).

DFT-D calculations correctly describe the existence of the new stable polymorph and indicate that no appreciable difference in the indirect bandgap (Fig. 4b–d) exists between the two polymorphs, suggesting that the large shift in the light absorption onset is instead related to a change in the fundamental photoexcitations in the two structures^27^,^28^. In β-coronene it is difficult to identify sharp resonances from Frenkel excitons as observed for the γ-polymorph. Instead, the structureless absorption band of the β−polymorph originates from charge transfer excitons that are favoured by the larger overlap between the molecular π orbitals. Moreover, the transition dipole moment of a charge transfer exciton is likely to be oriented parallel to the *a–b* plane, which would ensure optimal coupling with the electromagnetic radiation in our experiment.

### Structural stability

The new β-polymorph structure is notable for the large change in the herringbone angle. We can gain insights into the relative stability of the β-coronene structure when compared with the γ-polymorph, by consideration of these changes in the CH⋯π hydrogen bonding motif. It is known that the stronger the hydrogen bond, the stronger the trend for linearity^29^,^30^ depending on the strength of the proton donor. From XRD, we can see that the CH⋯π angle in γ-coronene of 95.86° suggests a strong, almost linear CH⋯π hydrogen bond, whereas in β-coronene, this angle becomes 49.71° (viz. Fig. 2). This change in angle would suggest a weakening of the hydrogen bonding in β-coronene, which is concomitant with the change in estimated CH⋯π hydrogen bond length^31^ that increases from 2.5 Å in the γ-polymorph to 3.0 Å in the β-polymorph. This weakening of the CH⋯π hydrogen bonding should therefore manifest itself as a diminution of the physicomechanical properties of β-coronene. To confirm this, we have determined the melting point (*T*_*M*_) and elastic modulus (*E*—the modal value measured on the (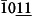 and 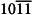 crystal faces) of both polymorphs and find that in γ-coronene, *T*_*M*_=436.14±0.01 °C and *E*=227 GPa, whereas for β-coronene *T*_*M*_=435.48±0.01 °C and *E*=92 GPa (Supplementary Figs 6 and 7). These data suggest weaker intermolecular forces in β-coronene at room temperature.

**Figure 5:**
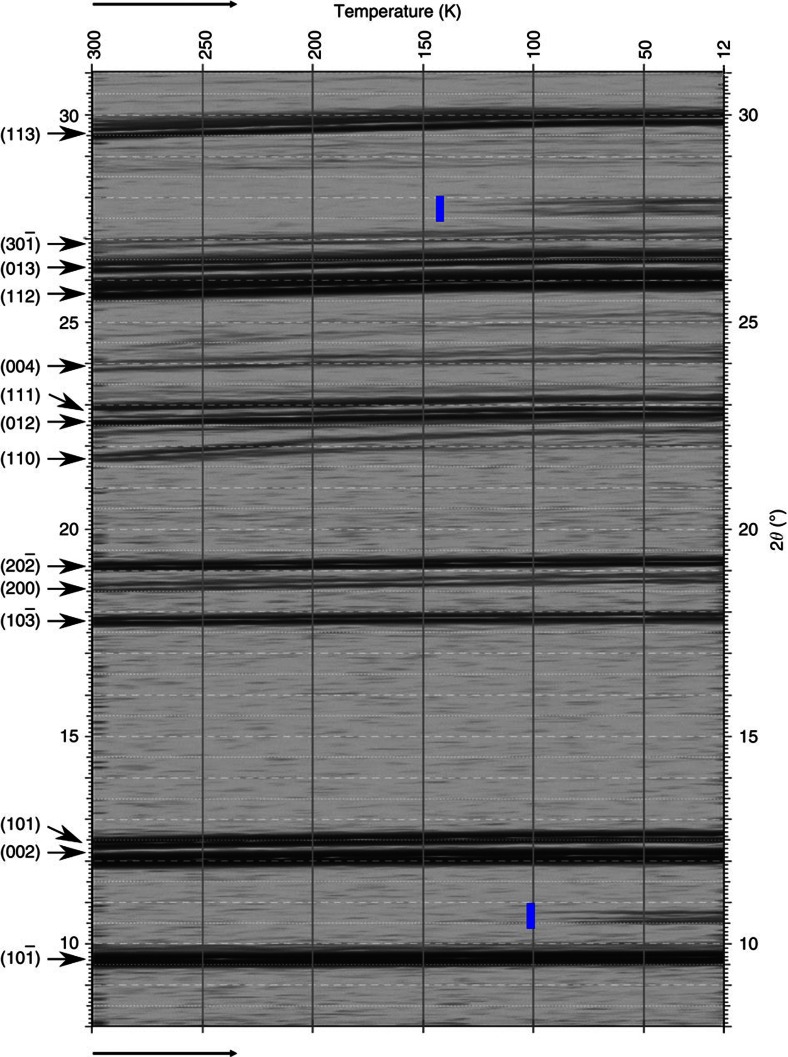
2D map of XRD peak shifts as a function of temperature in γ-coronene. Temperature is decreased from 300 to 12 K (left to right). Blue markers indicate the emergence of the new peaks due to β-coronene formation. The blue line near 10° 2*θ* encompasses two emergent peaks.

DFT-D calculations show that β-coronene is actually the more stable of the two polymorphs at 0 K, by 3.8 kJ mol^−1^ based on lattice energy differences (Supplementary Table 1). Confirmation that this is the case comes from powder XRD of polycrystalline γ-coronene recorded through a temperature cycle from room temperature to 12 K. On cooling through a temperature of 150 K, three new peaks emerge which cannot be indexed to the γ−polymorph (JCPDS card number 12-1611; Supplementary Fig. 8). These emergent peaks correspond to the (002), (101) and (112; 2*θ*=10.55°, 10.67° and 27.72° respectively) reflections of the new polymorph, β-coronene. Thus β- and γ-coronene are enantiotropic polymorphs, with a critical temperature between 100 K and 150 K (Fig. 5). In general, in an enantiotropic pair of polymorphs, the more intrinsically stable member will have the greater specific enthalpy of fusion. As the differential scanning calorimetry (DSC) experiment to determine melting point (Supplementary Fig. 6) is conducted at far higher temperatures than 150 K, it is the γ-coronene polymorph which will be the more stable at these temperatures and therefore be expected to have the greater specific enthalpy of fusion. From Supplementary Fig. 5, we can calculate the values for the heat of fusion for each of the polymorphs as 20.78 kJ mol^−1^ and 25.26 kJ mol^−1^ for β- and γ-coronene, respectively, confirming that these are enantiotropic polymorphs.

In summary, through judicious application of a magnetic field, we have demonstrated that a well-studied PAH can be grown as a new polymorph and confirmed that a range of physical properties have been significantly altered. The optical properties of the β-coronene crystal have been transformed to such an extent that it absorbs panchromatically from the UV into the near-IR. This new single crystal may therefore be of great interest in organic optoelectronics and photovoltaics.

## Methods

### Coronene

Coronene crystals (Sigma Aldrich, purity 97%) were recrystallized from toluene and purity assayed by nuclear magnetic resonance (C^13^ and H^+^). Trace metals were tested for using inductively coupled plasma—atomic emission spectroscopy (Agilent 710 ICP-AES) after digestion in 3% nitric acid (Sigma Aldrich, ≥99.999%, trace metal basis). Structural analysis was conducted by optical microscopy and single crystal XRD. Analysis of the single crystal data of non-magnetic field grown crystals suggests favourable growth along the *b*-axis, which form the characteristic needle shaped crystals.

### Crystal growth under 1 T of magnetic field

A supersaturated solution of coronene (2.5 mg ml^−1^) in toluene was prepared and stored in an oven at 93 °C. The solution was then passed through a 0.22-μm PTFE filter directly into a 5-mm quartz cuvette with a stopper. Once sealed, the cuvette was placed in the magnetic cavity (Supplementary Fig. 9) and the whole system maintained at 93 °C for 4 h post cuvette insertion. The oven was then programmed to cool to 83, 73 and 63 °C then finally 50 °C, with a 24-h dwell at each temperature, before cooling to room temperature. Crystals of β-coronene are the sole polymorph to grow under the magnetic field and to-date the experiment has been repeated >10 times.

### Single crystal X-ray crystallography

Intensity data for all coronene structures were collected at different temperatures on an Agilent SuperNova-E Dual diffractometer equipped with an Oxford Cryosystem, using CuKα radiation (*λ*=1.5418 Å). Data were processed using the CrysAlisPro software (CrysAlisPro, Agilent Technologies, Version 1.171.37.35 (release 13-08-2014 CrysAlis171 .NET; compiled on 13 Aug 2014). For all structures a symmetry-related (multi-scan) absorption correction was applied. Crystal parameters are provided in Supplementary Table 2. See also Supplementary Data 1 and 2. Structure solution, followed by full-matrix least squares refinement was performed using the WINGX-v2014.1 suite of programs throughout.

### Physical characterization

Optical absorption spectra were recorded with a ultraviolet–visible–near infrared spectrometer Agilent Cary 5000 measuring in transmission configuration. The spectrometer can measure absorbance up an optical density of 10. Single crystals of β- or γ-coronene were suspended in the sample beam path. Unpolarized light was shone perpendicular to the *a–b* plane of the unit cells. High-quality crystals with flat surfaces were carefully selected to avoid light scattering effects. The beam size was narrowed to half the lateral size of crystals. All absorption spectra were recorded at room temperature in air. Elastic moduli were determined using an Asylum Research MFP-3D Infinity AFM operating in AMFM viscoelastic imaging mode, using an AC160TS-R3 silicon tip (9±2 nm radius). Freshly cleaved mica was used as a calibration standard (measured at 178 GPa). Melting point determination was done using a TA-Instruments Q100 DSC with temperature ramp of 1 °C min^−1^ between 35 and 460 °C with 2.2 mg of coronene hermetically sealed under a nitrogen atmosphere in an aluminium pan.

### Computational calculations

Computational calculations have been performed with density functional codes CASTEP (ref. 32) and VASP (ref. 33), using the Perdew–Burke–Enzerhof exchange correlation functional with semi-empirical dispersion corrections (DFT-D) to account for van der Waals interactions. CASTEP calculations (version 7.03) used in-built ultra-soft pseudopotentials for C and H atoms, a plane wave cutoff of 600 eV, and Monkhorst-Pack^34^ k-point samplings of 3 × 4 × 2. Cell parameters and atomic coordinates were fully relaxed using the Broyden–Fletcher–Goldfarb–Shanno (BFGS) method, halting when residual forces fell below 1 meV Å^−1^. Changing the cutoff energy from 450 to 600 eV caused structural parameters to change by <0.001 nm or <0.01°. Increasing k-point sampling to 6 × 8 × 4 changed energies by <1 meV. Robustness of results to choice of semi-empirical dispersion correction was assessed through the use^35^ of using the Grimme scheme^36^ with both default vdW radii (*R*_H_=1.001, *R*_C_=1.452) and experimental (*R*_H_=1.090, *R*_C_=1.750), as previously employed by Fedorov *et al*.^37^ for γ-coronene. VASP calculations (version 5.3.3) use PAW potentials^38^ with 500 eV energy cutoff, 3 × 4 × 2 k-point sampling and the Tkatchenko-Scheffler^39^,^40^ dispersion correction. Geometry optimization was performed starting from experimental cell parameters and atom coordinates, aboth with and without symmetry constraint. Stability of optimized geometries was verified by re-optimizing after randomly displacing atoms by 0.05 Å in *x*, *y* and *z*.

### Data Availability

Original optical images and raw data from XRD, AFM and DFT-D calculations pertaining to all materials in this manuscript have been placed in the University of Bristol Research Data Repository (https://data.bris.ac.uk/):

Windows path: \\rdsfcifs.acrc.bris.ac.uk\Polymorphism_in_coronene

Linux path: smb://rdsfcifs.acrc.bris.ac.uk/Polymorphism_in_coronene

Mac OSX path: smb://rdsfcifs.acrc.bris.ac.uk/Polymorphism_in_coronene.

## Additional information

**Accession codes**: The X-ray crystallographic coordinates for structures reported in this Article have been deposited at the Cambridge Crystallographic Data Centre (CCDC), under deposition number CCDC 1409823. These data can be obtained free of charge from The Cambridge Crystallographic Data Centre via www.ccdc.cam.ac.uk/data_request/cif.

**How to cite this article**: Potticary, J. *et al*. An unforeseen polymorph of coronene by the application of magnetic fields during crystal growth. *Nat. Commun.* 7:11555 doi: 10.1038/ncomms11555 (2016).

## Supplementary Material

Supplementary Information Supplementary Figures 1-9 and Supplementary Tables 1-2

Supplementary Data 1 Crystallographic information file for gamma coronene

Supplementary Data 2 Crystallographic information file for beta coronene
