# Supplementary material for: An unforeseen polymorph of coronene by the application of magnetic fields during crystal growth
Source: Nat Commun. 2016 May 10;7:11555. doi: 10.1038/ncomms11555 (PMC4866376; doi:10.1038/ncomms11555)
Supplement: Supplementary Information — Supplementary Figures 1-9 and Supplementary Tables 1-2 [file ncomms11555-s1.pdf]

Supplementary Information

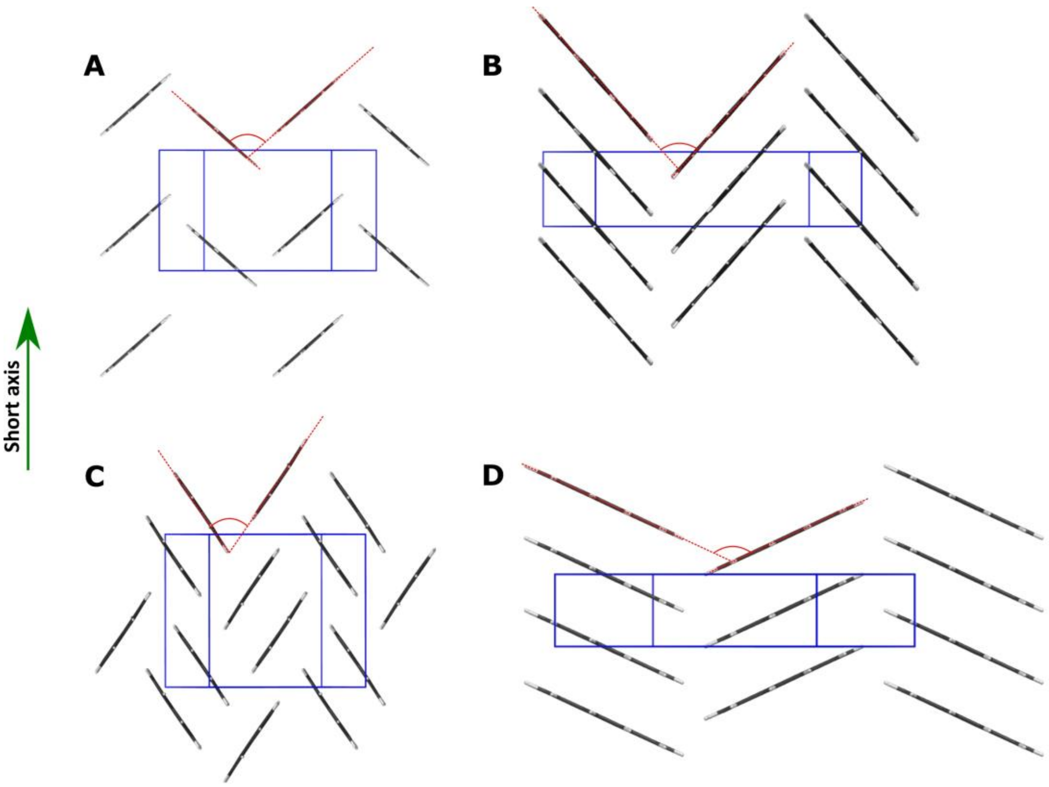

Supplementary Figure 1. The four classes of crystal packing in polyaromatic hydrocarbons. (A) is the herringbone (HB) structure, (B) the gamma-herringbone (γ-) structure, (C) the sandwich-herringbone (SHB) structure and (D) the beta-herringbone (β-) structure.

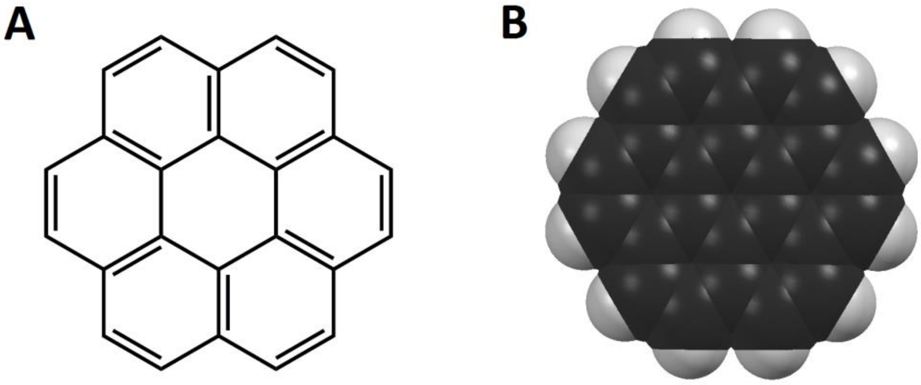

Supplementary Figure 2. The molecular structure of coronene shown as (A) skeletal and (B) space-filling models.

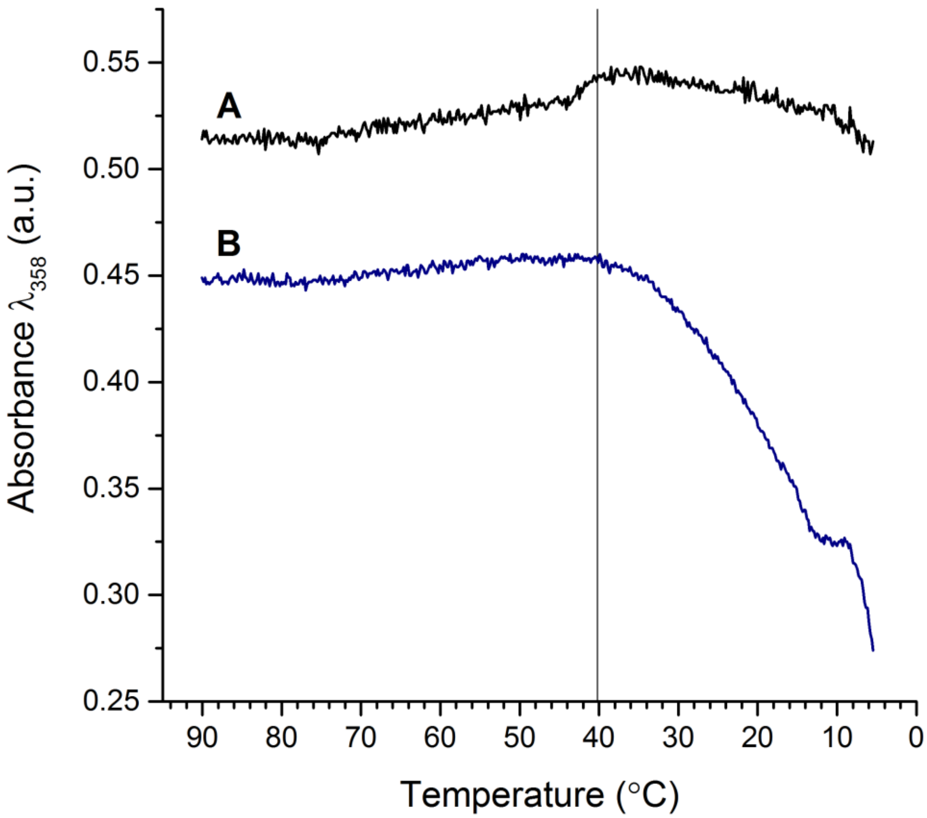

Supplementary Figure 3. UV-vis absorbance at 358 nm as a function of temperature for coronene crystallising under (A) 1 T and (B) 0 T. The clear decrease in absorbance at 40 °C in the 0 T experiment is indicative of crystal growth at this temperature.

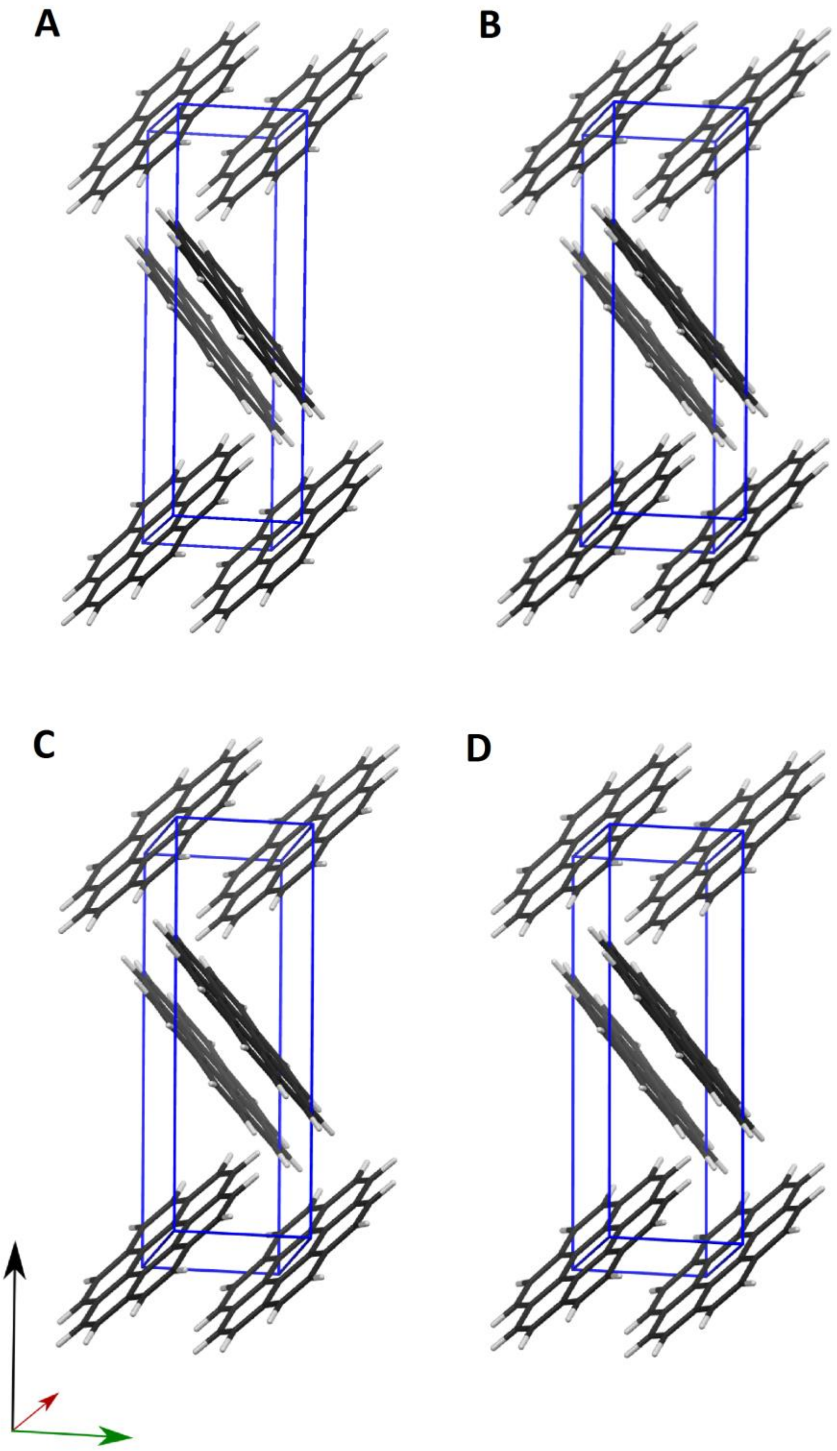

Supplementary Figure 4. X-Ray diffraction crystal structures of coronene grown under a range of applied fields. In (A) the applied field was 0 T; in (B) 0.2 T; in (C) 0.5 T and in (D) 0.8 T. Red green and black arrows indicate the direction of the *a*-, *b*- and *c*-axis respectively.

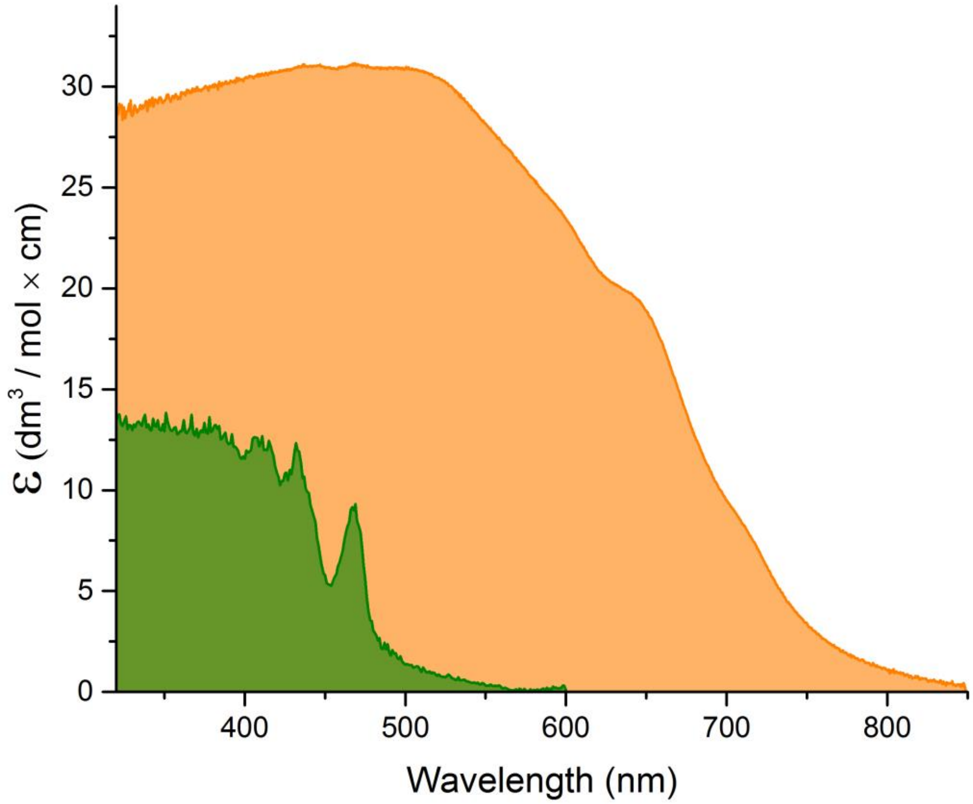

Supplementary Figure 5. Absorption spectra of γ-coronene (green) and β-coronene (orange) single crystals plotted as the extinction molar coefficient (epsilon) vs wavelength. Unpolarized light was irradiated perpendicular to the *a-b* plane at room temperature.

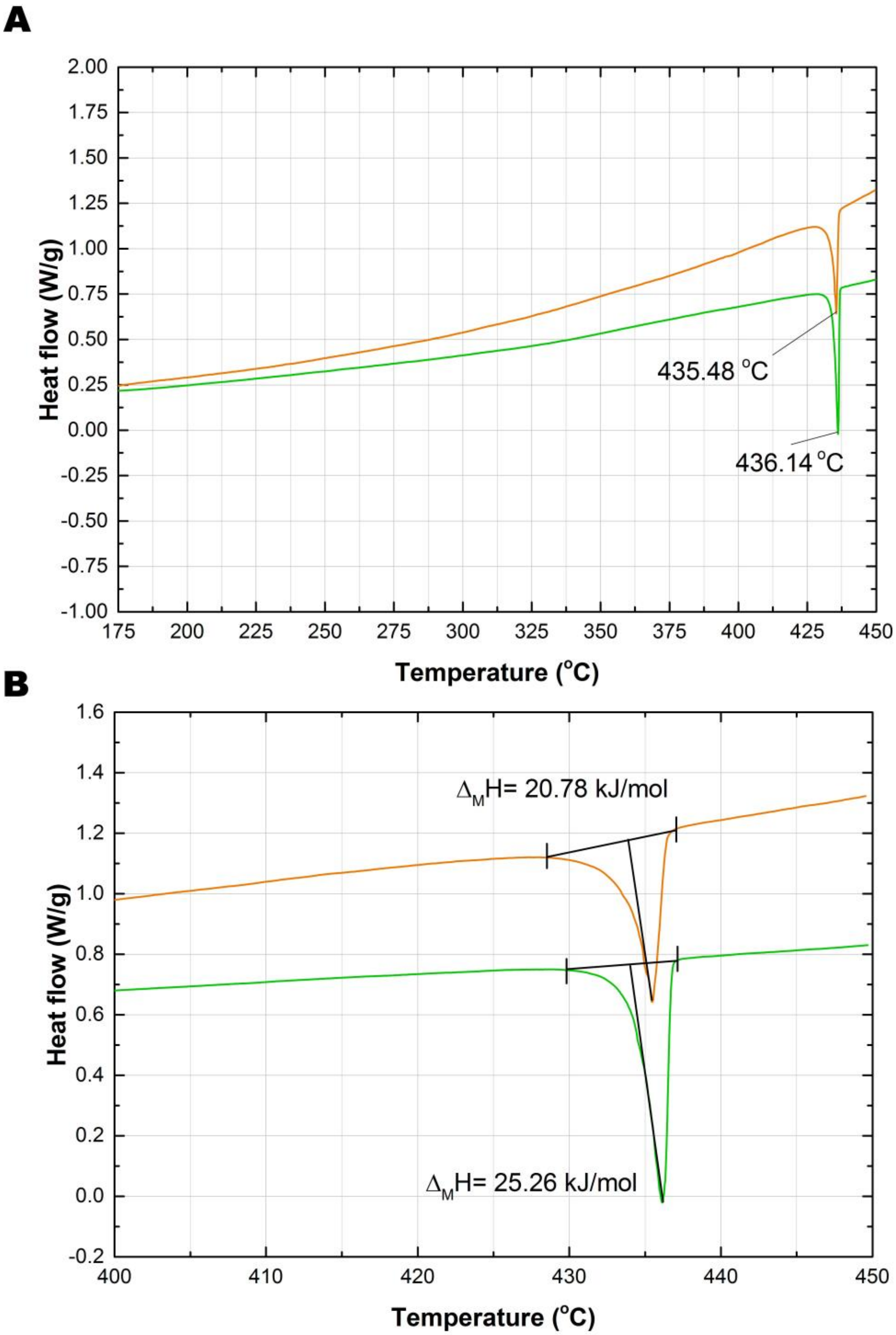

Supplementary Figure 6. DSC curves showing the melting profile of β-coronene (orange) and γ-coronene (green). In (A) the melting points are highlighted and in (B) the values of enthalpy of fusion.

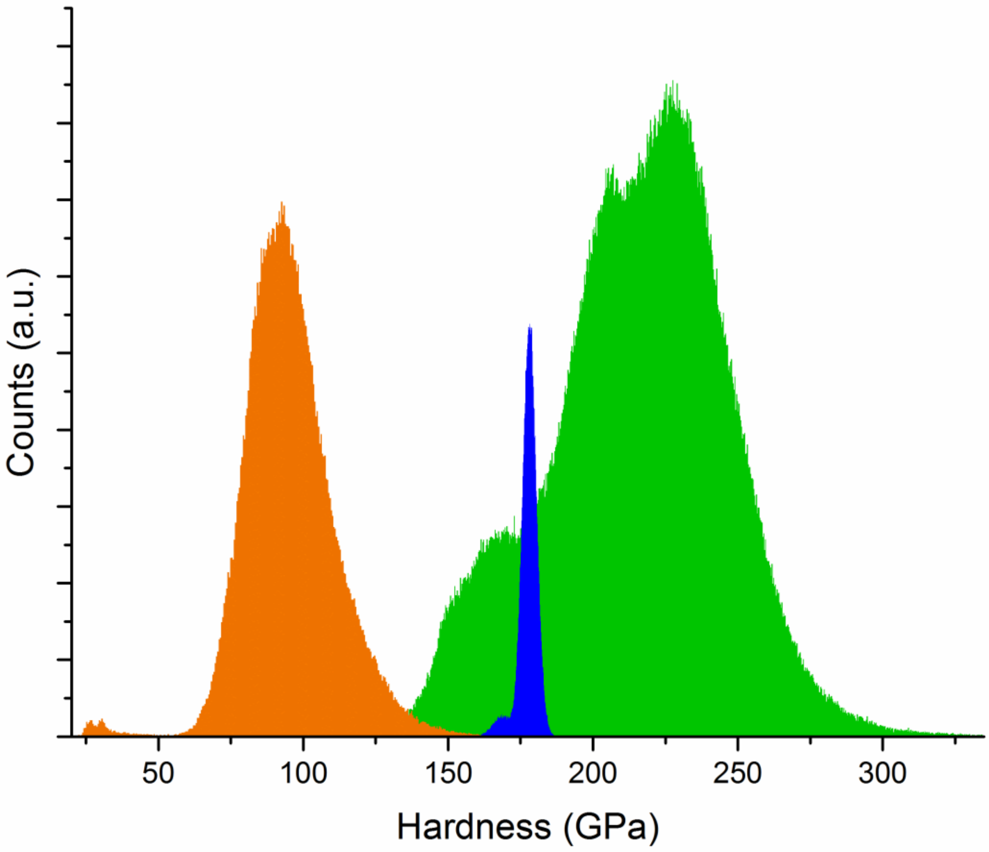

Supplementary Figure 7. Elastic moduli measured via AFM. The orange data are the results from β-coronene, the green from γ-coronene and the blue from a mica control sample. Coronene single crystals were measured on the $\bar{1}0\underline{11}$ and $10\underline{\overline{11}}$ crystal faces.

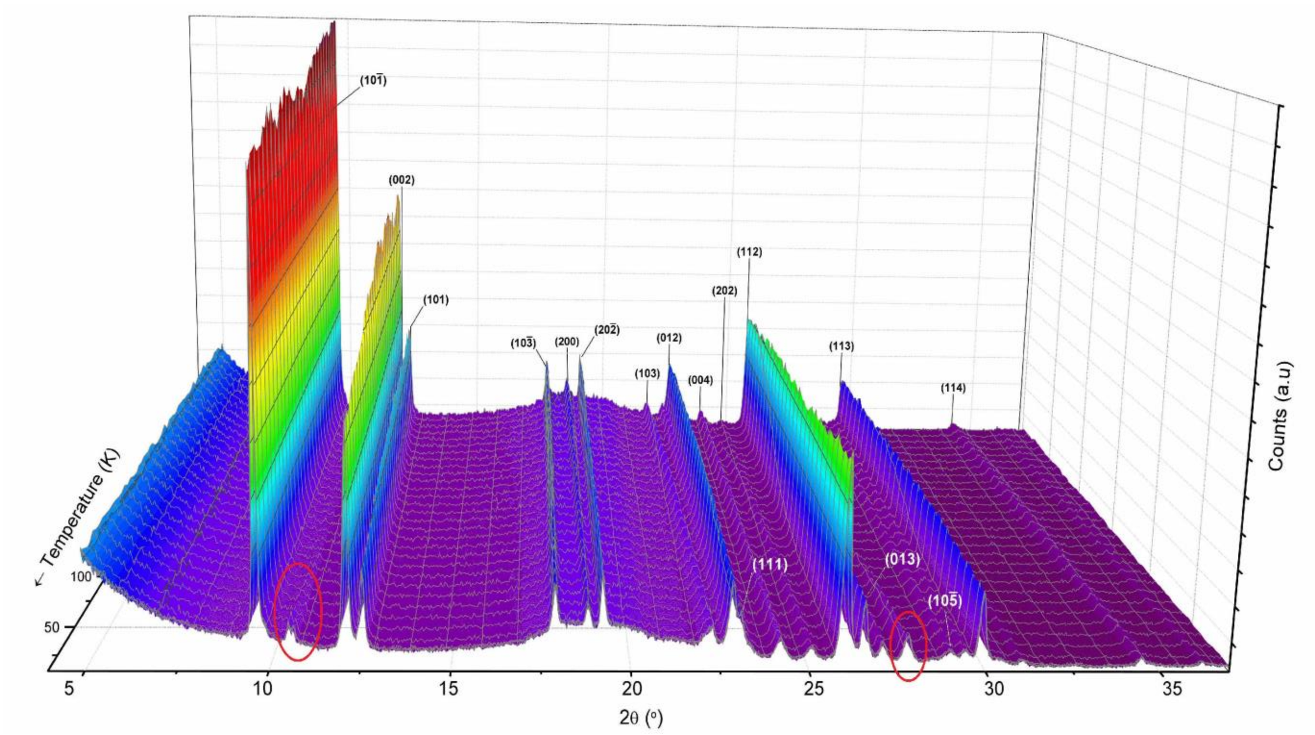

Supplementary Figure 8. 3D surface plot of powder XRD as a function of temperature for γ-coronene while cooling from 300 K to 12 K, without background correction. Arrow indicates forward direction of temperature. Color gradient is arbitrary used only for assisting with visualizing intensities. Red ovals identify emergent peaks due to the formation of β-coronene (viz. Fig. 5). The red oval near 10° 2θ encompasses two emergent peaks.

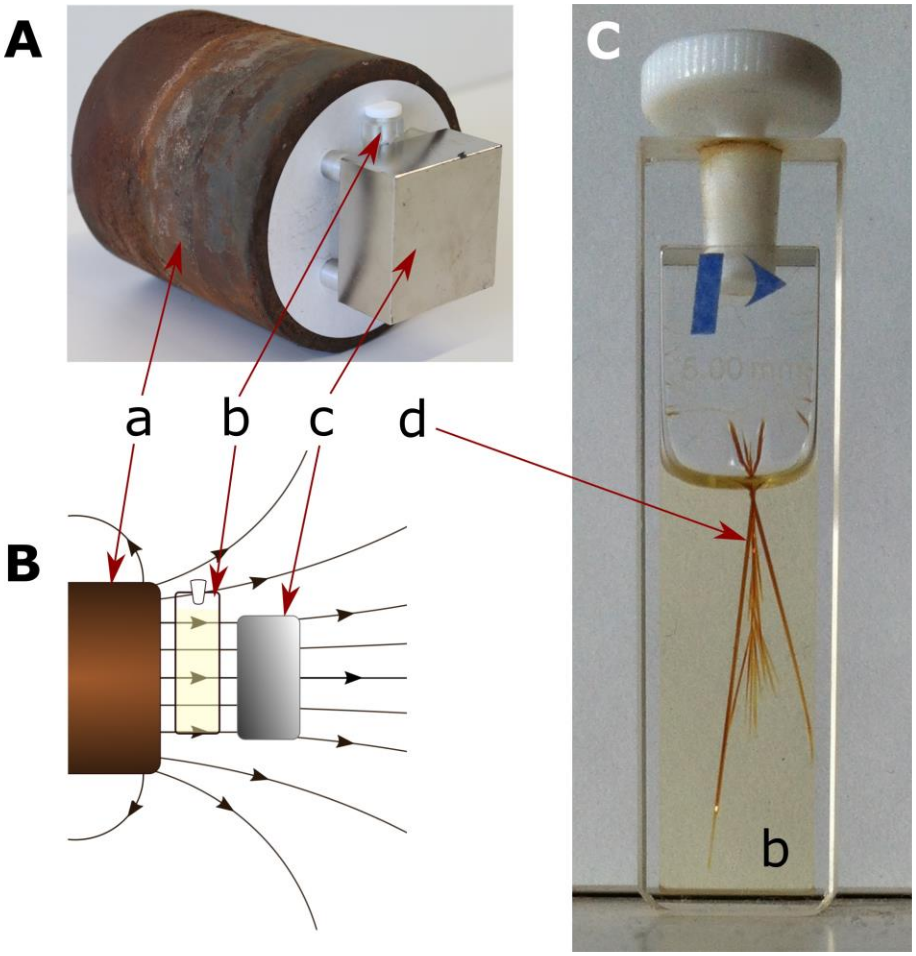

Supplementary Figure 9. Experimental configuration for the growth of β-coronene. (A) Optical image of the experiment, (B) schematic and (C) reaction vial with crystals of β-coronene *in-situ*. (a) is the 1 T magnet; (b) the sample vial; (c) the magnetic holding plate and (d) crystals of β-coronene. Field lines from the 1 T magnet through the experiment are indicated. Field strength was controlled by the application of paper shims (200 μm thickness) between the magnet and the sample vial. The magnetic field was quantified using a Model 5170 gaussmeter from F. W. Bell.

Code	vdW	form	*a* [Å]	*b* [Å]	*c* [Å]	β [°]	*d* [Å]	∠ [°]	*V* [Å^3]	E_γ-E_β [kJ/mol]
CASTEP	G06[a]	γ	9.779	4.548	15.422	107.26	3.29	87.4	655	4.3
		β	10.254	3.672	16.851	95.76	3.35	48.5	631	
CASTEP	GF[b]	γ	9.924	4.665	15.915	105.53	3.52	82.1	710	3.8
		β	10.336	3.871	17.264	95.04	3.58	44.9	688	
VASP	TS[c]	γ	9.954	4.566	15.443	106.57	3.35	85.6	673	3.4
		β	10.319	3.714	17.253	95.86	3.39	48.1	658	

[a] Grimme (2006) – ref. (*35*).
[b] Grimme (2006) with experimental van der Waal radii as suggested by Fedorov *et al* (*36*).
[c] Tkatchenko-Scheffler – refs. (*38, 39*).

Supplementary Table 1. Calculated structural parameters for coronene in the two crystal forms γ and β. Constructed from DFT-D calculations using codes and dispersion corrections (vdW) as indicated. Lengths *a*, *b*, *c* are the lattice parameters and β the monoclinic angle; *d* is the interplanar distance between parallel coronene molecules. The columns labelled ∠ and V report the herringbone angle between adjacent coronene molecules and the unit cell volume, respectively. The final column reports the lattice energy difference between the two structures; positive indicates β is the more stable.

Form	Temp [K]	Space Group	***a* [Å]**	***b* [Å]**	***c* [Å]**	**β[°]**	**d[Å]**	**∠ [°]**	**V [Å^3]**
γ	150	$P2_1/n$	10.02	4.67	15.06	106.7	3.43	-	699
γ	200	$P2_1/n$	10.040	4.681	15.6041(19)	106.32	3.43	85.76	703.77
γ	250	$P2_1/n$	10.072(3)	4.6907(12)	15.650(6)	106.18	3.44	85.59	710.1(4)
β	80	$P2_1/n$	10.386	3.821	17.211	96.24	3.47	49.7	679
β	150	$P2_1/n$	10:392	3.839	17.229	96.24	3.48	50.0	683

Supplementary Table 2. Experimental structural parameters for coronene in the two crystal forms γ and β. The parameters *a*, *b*, *c* are the three unit cell axis and β the monoclinic angle, d represents the interplanar distance between parallel coronene molecules. The columns labelled ∠ and V report the herringbone angle between adjacent coronene molecules and the unit cell volume, respectively.
